# Conserved Partial Reprogramming Effects on the Methylome

**DOI:** 10.3390/epigenomes10030055

**Published:** 2026-08-13

**Authors:** Elena-Cristina Găitănaru, Roua Gabriela Popescu, Andreea-Angelica Stroe, Sergiu Emil Georgescu, George Cătălin Marinescu

**Affiliations:** 1 University of Bucharest, Faculty of Biology, Splaiul Independentei 91-95, R-050095 Bucharest, Romania; cristina.gaitanaru@independent-research.ro (E.-C.G.); angelica-andreea.stroe@bio.unibuc.ro (A.-A.S.); sergiu.georgescu@bio.unibuc.ro (S.E.G.); 2 Independent Research Association, 012416 Bucharest, Romania; 3 Blue Screen SRL, 012416 Bucharest, Romania

**Keywords:** epigenetic rejuvenation, partial reprogramming, Yamanaka factors, DNA methylation integrative secondary analysis, anti-aging therapy

## Abstract

Background/Objectives: Among the many proposed theories of aging, a growing body of research points to epigenetic alterations, particularly changes in DNA methylation patterns, as key contributors to biological decline. DNA methylation can be targeted and modulated to induce anti-aging and regenerative effects using an emerging therapeutic approach known as partial reprogramming, induced by different combinations of Yamanaka factors. Methods: In this study, we performed an integrative secondary analysis of five publicly available DNA methylation datasets derived from partial reprogramming experiments across multiple mammalian species, tissues, cell types and combinations of transcription factors (*n* = 189 samples). Results: Following differential methylation analysis of 929,009 CpGs, we identified 14 CpG sites showing reproducible and significant changes, with two CpGs consistently present across all datasets. Conclusions: These findings suggest that partial reprogramming may exert anti-aging and regenerative effects, in part, through the selective modulation of conserved DNA methylation sites.

## 1. Introduction

Aging is characterized by progressive functional decline, loss of tissue homeostasis and accumulation of molecular damage [1,2,3,4]. Among the many proposed biomarkers of aging, epigenetic modifications, such as changes in DNA methylation patterns, have emerged as robust indicators of biological age [5,6,7,8,9]. Over the past decade, the expression of different combinations of pluripotency transcription factors (OCT4, SOX2, KLF4, c-MYC, LIN-28, and NANOG, collectively referred to as OSKMLN) have been reported to promote regeneration of age-associated phenotypes [10,11,12,13,14] and to reverse the epigenetic clock signature by up to 30 years in human dermal fibroblasts (HDFs) [15,16] without inducing complete dedifferentiation [17,18,19]. These findings have given rise to the concept of partial reprogramming as a promising anti-aging strategy, currently being translated into clinical trials as a targeted therapy for age-related diseases, such as glaucoma [20].

Mechanistically, partial reprogramming is often discussed in terms of transcription factor activity and chromatin remodeling [21]. These six transcription factors enter the nuclei of somatic cells and are thought to bind to specific genomic regions called pluripotency-associated recognition sequences [22]. They subsequently reshape chromatin accessibility through coordinated interactions with epigenetic modifications [22,23], developmental signaling pathways [24] and ATP-dependent chromatin remodelers [25,26]. Much of the scientific literature has, therefore, focused on how these factors interact with DNA or with each other to alter the enhancer–promoter landscape and modulate transcription programs associated with pluripotency, plasticity, and regeneration [27,28,29,30,31]. Although chromatin dynamics, transcriptional rewiring and epigenetic age reversal have been extensively studied, it is important to note that cells, tissues, and different mammalian species appear to respond differently to pluripotency-associated transcription factor exposure [32,33,34]. Variations in delivery methods, duration of expression, and factor combinations (OSK, OSKM, and OSKMLN) further contribute to heterogeneous outcomes [18,34,35]. Some cell types exhibit measurable rejuvenation of age-related phenotypes, whereas others show a limited response [19,36]. This heterogeneity raises a fundamental question: are there specific regions in the methylome that respond consistently to partial reprogramming with these transcription factors, regardless of species, tissue type, or treatment protocol?

Identifying such methylation markers would provide insight into whether partial reprogramming induces its anti-aging effects through global epigenetic modulation or through selective modulation of key regulatory loci. Evolutionarily conserved CpG sites across mammalian genomes could represent stable epigenetic signatures of factor activity and serve as biomarkers for monitoring partial reprogramming as a future systemic anti-aging intervention.

To our knowledge, no previous study has addressed this question; therefore, we conducted an integrative cross-dataset analysis of publicly available DNA methylation datasets derived from previous partial reprogramming experiments. By integrating data across three mammalian species, multiple tissues, cell types and treatment conditions, we aimed to identify conserved and reproducible methylation signatures associated with partial reprogramming despite substantial biological heterogeneity.

## 2. Results

**Figure 1:**
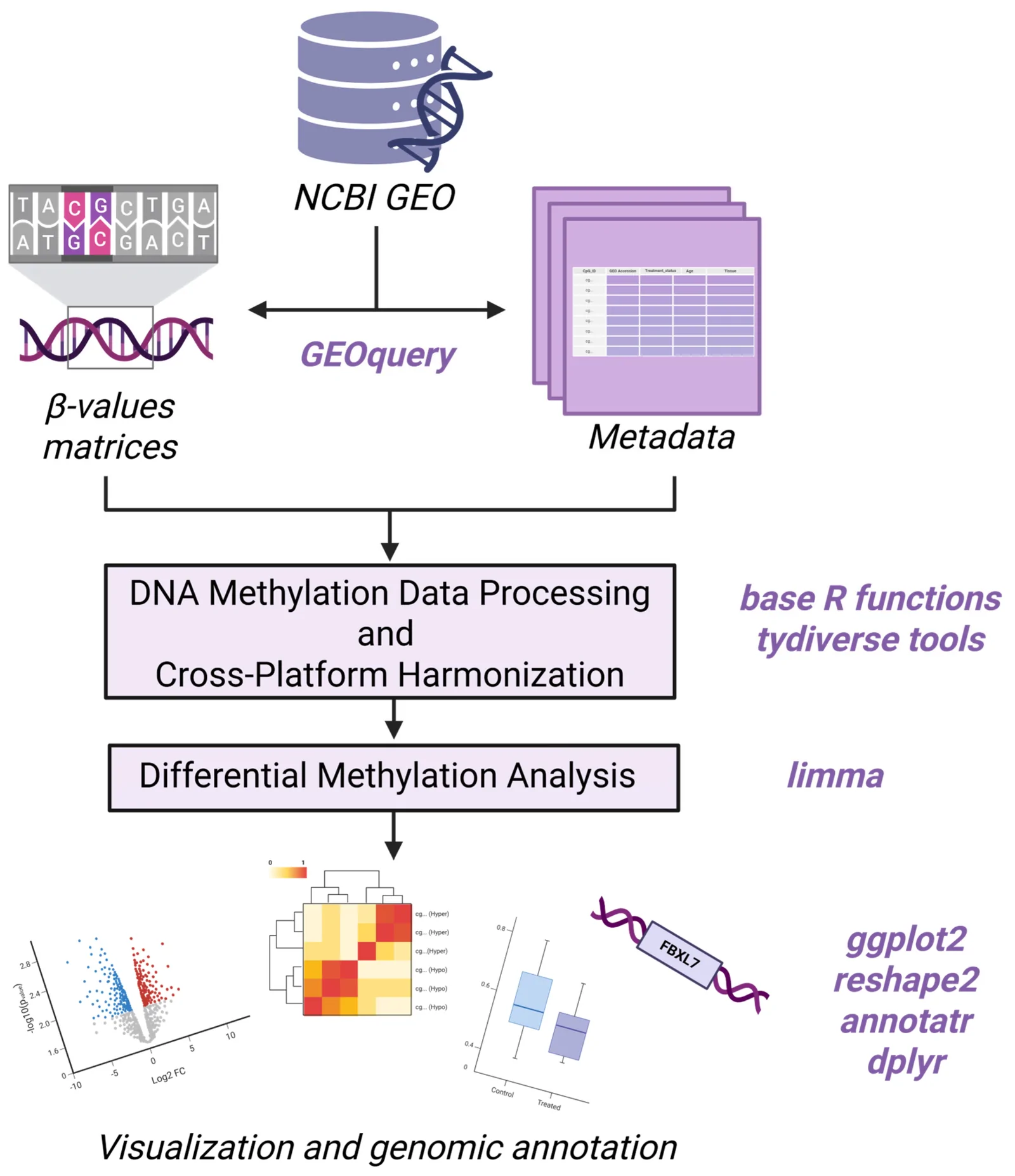
DNA methylation integrative secondary analysis workflow diagram (created in https://BioRender.com).

In this study, we performed an integrative secondary analysis of five publicly available DNA methylation datasets derived from partial reprogramming experiments reporting anti-aging effects (Figure 1) [10,16,19,33,35,37]. In total, 189 samples obtained from the Gene Expression Omnibus (GEO), comprising treated (OSK, OSKM and OSKMLN) and corresponding controls, were included in the analysis. After conversion of β-values to M-values and batch adjustment using dataset-specific covariates, differential methylation analysis was conducted using the limma framework in R. Across the different array platforms, a total of 929,009 CpGs were identified in the union dataset. Owing to the high proportion of missing data across arrays, CpGs present in at least 80% of samples (37,554 CpGs) were retained for downstream analysis. We identified 14 CpGs showing significantly different methylation levels (DMCs) (*adj.p.Val* < 0.05; |log_2_FC| > 0.5) between treated (OSK, OSKM or OSKMLN) and control samples (Supplementary Material S1, Tables S1 and S2). Of these, four CpGs were shared across all analyzed genomes, tissues and treatment conditions. The remaining ten CpG sites were present only in rat and mouse genomes and were specific to OSK and OSKM treatment conditions. After annotation, 12 of these CpGs were mapped onto known genomic loci and further characterized (Supplementary Material S1, Table S3).

### 2.1. Dataset Characteristics

Five publicly available DNA methylation datasets (GSE252401, GSE142439, GSE147436, GSE190665, and GSE54848) were included in this cross-platform integrative secondary analysis (Table 1). All datasets were obtained from GEO and generated using Illumina’s HumanMethylation450 BeadChip (HM450K), MethylationEPIC BeadChip (EPIC) or Horvath’s pan-mammalian methylation array (Mammal40k). The original studies included human, mouse, and rat samples derived from multiple tissues and cell types under control and partial reprogramming conditions with different combinations of pluripotency-associated transcription factors (OSK, OSKM, and OSKMLN). Each study included distinct experimental designs and treatment regimens to safely induce partial cellular reprogramming.

The GSE252401 dataset originates from the study by Horvath et al. [10], which contains the DNA methylation frequencies measured after 39 days of continuous OSKM expression. The Sarkar et al. [35] dataset (GSE142439) consists of methylation assessed two days after a short four-day OSKMLN treatment. Similarly, the dataset from Lu et al. [19] (GSE147436) consists of DNA methylation data measured after a short period of OSK expression, only five days. The Browder et al. [33] dataset (GSE190665) contains data assessed after a long-term (7–10 months) cyclic (two days on/five days off) OSKM partial reprogramming protocol. The Ohnuki et al. [37] dataset (GSE54848) contains DNA methylation measurements across multiple reprogramming stages from day 0 to day 49 of OSKM expression. To ensure inclusion of only partial reprogramming states, we followed Olova et al.’s [16] article and chose only the measurements from days 7 and 11. Collectively, these datasets represent a spectrum of partial reprogramming states, ranging from transient early responses to prolonged and cyclic induction conditions. Although the experimental designs differ, all datasets were interpreted within a shared partial reprogramming framework.

**Figure 2:**
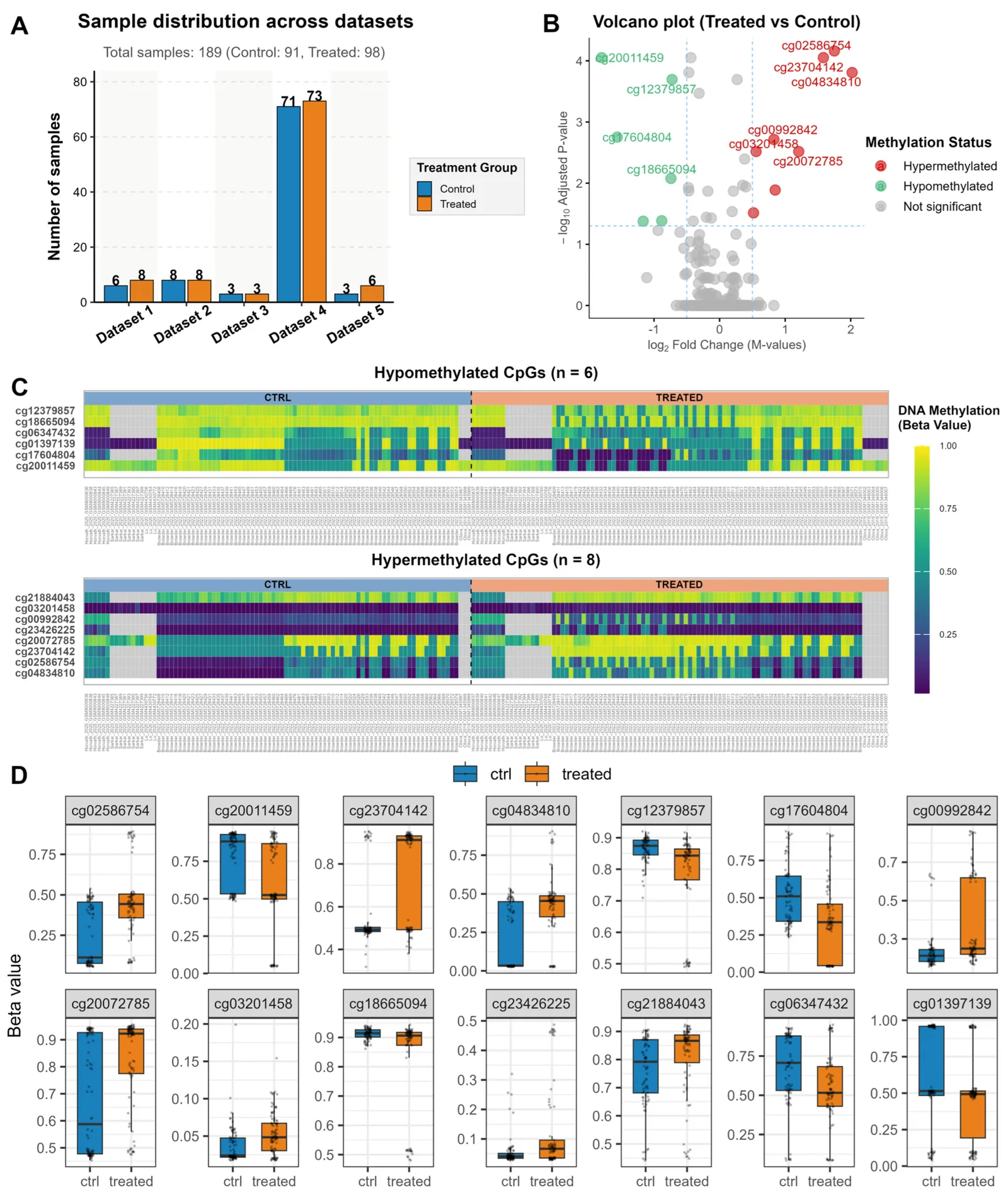
Differential methylation associated with partial reprogramming. (**A**) Sample distribution displaying the number of control and treated samples across the five datasets included in the integrative secondary analysis. (**B**) Volcano plot showing log_2_(fold changes) versus statistical significance −log_10_(adjusted *p*-value) for all analyzed CpGs. Hypermethylated CpGs are highlighted in red, hypomethylated in green, and non-significant in grey. (**C**) Heatmaps of the hypomethylated and hypermethylated significant CpGs across all 189 samples, showing consistent methylation patterns between treated and control groups. Hypermethylated sites are in yellow, hypomethylated in dark blue. (**D**) Boxplots of β-values for each significant CpG site, displaying individual sample β-values as jittered points.

For each dataset, β-values matrices and corresponding metadata were organized to retain only samples meeting predefined inclusion criteria (old controls and treated samples undergoing partial reprogramming). Samples not fulfilling these criteria were excluded. After filtering and harmonization across methylation arrays, 189 samples were retained for downstream analysis (Figure 2A).

To ensure cross-platform compatibility, CpGs were unified using the union of Illumina probe IDs present across datasets (929,009 CpGs). Sites with excessive missing values (>20%) were removed. The final matrix comprised 37,554 CpGs across 80% of samples.

### 2.2. Cross-Platform Integrative Secondary Analysis Identifies Limited Differential Methylation

Differential methylation analysis was performed using limma’s linear modeling framework with empirical Bayes moderation. After correction for multiple testing (*adj.p.Val* < 0.05), a total of 14 CpG sites were identified as significantly associated with the partial reprogramming treatment (Supplementary Material S1, Tables S1 and S2). As a robustness analysis, we intersected all five datasets and retained only CpGs without missing values. Following limma and significance filtering, only two CpGs (cg20011459 (*adj.p.Val* = 0.0000598; |log_2_FC| = 1.859) and cg01397139 (*adj.p.Val* = 0.0367; |log_2_FC| = 1.212)) remained significant, corresponding to the first two significant CpGs in the union analysis (*adj.p.Val* < 0.05) (Supplementary Material S1, Table S4). Despite the integration of multiple independent datasets and platforms, the number of significant CpGs was limited, which is consistent with the possibility that partial reprogramming induces selective rather than genome-wide methylation remodeling.

A volcano plot was generated to visualize the statistically significant differential methylation results (Figure 2B). The *x*-axis represents the log fold change (log_2_FC), with positive values indicating hypermethylation and negative values indicating hypomethylation. The *y*-axis shows the -log10 of the adjusted *p*-value (*adj.p.Val*), highlighting the statistical significance of each CpG site. The 14 CpG sites that met the significance thresholds (*adj.p.Val* < 0.05; |log_2_FC| > 0.5) were highlighted according to their methylation status: hypermethylated sites in red, hypomethylated sites in green, and non-significant sites in grey. In total, eight CpGs were identified as hypermethylated, whereas six were hypomethylated, demonstrating distinct patterns of gain and loss of DNA methylation in response to partial reprogramming.

To visualize treatment-specific methylation patterns, a heatmap was generated using the β-values of significant CpGs (Figure 2C). Hierarchical clustering revealed clear separation between treated and control samples, indicating consistent directionality of methylation changes across datasets regardless of treatment, species, tissue or cell type. Boxplots of the top differentially methylated CpGs further confirmed reproducible differences in β-values between groups (Figure 2D). These loci demonstrated a consistent direction of effect across samples despite cross-study heterogeneity.

### 2.3. Genomic Distribution, Functional Annotation and Evolutionary Conservation

Using chromosomal coordinates obtained from Illumina and Horvath’s Mammalian Methylation Consortium [38,39] annotations (hg19, rn6, and mm10), the 14 significant CpGs were distributed across multiple chromosomes without clustering in a single genomic region. Genomic annotation revealed that these CpGs were located within promoter regions, CpG islands, shores, gene bodies and intergenic regions, suggesting potential regulatory relevance. Of these, 12 CpGs were consistently annotated across platforms and retained for downstream functional interpretation. Two CpG sites (cg02586754 and cg21884043) lacked chromosomal annotation in the currently available rodent reference genomes and were, therefore, listed as *NA* (Table 2). Functional enrichment analysis of genes associated with the four CpG loci consistently identified across all analyzed species and cell types revealed significant enrichment of biological processes related to cell-cycle regulation, telomere maintenance, developmental patterning and lipid metabolism. A complete list of enriched Gene Ontology (GO) terms is provided in Supplementary Material S1, Table S5.

Evolutionary conservation analysis revealed that several of the 12 annotated CpG sites were located within evolutionarily constrained genomic regions. Human loci displayed PhyloP scores ranging from −0.22 to 4.84, whereas rodent loci exhibited scores ranging from −0.4 to 2.74. Overall, seven CpG loci (58.3%) were located within regions showing moderate to strong evolutionary constraint across vertebrate species (PhyloP > 1), while five CpG loci (41.6%) showed low conservation scores (PhyloP < 1). The complete list of PhyloP conservation scores for all analyzed CpG sites is provided in Supplementary Material S2.

### 2.4. Transcription-Factor-Binding Enrichment Analysis

To investigate potential transcription factor associations, ChIP-Atlas enrichment analysis was performed using genomic regions extending ±1 kb around the four CpG loci shared among all species and cell types included in the analysis. Several transcription factor datasets, including ERG, ZNF687, ASCL1, TRIM25, CDK9, ATF3, and KAT6A, presented overlap with two or three queried regions. However, none of the identified transcription factor datasets reached statistical significance following multiple-testing correction (log *Q-value* = 0). Isolated overlaps with ChIP-seq datasets for reprogramming-associated factors, including KLF4, MYC, and NANOG, were identified. However, these datasets originated from cellular models that were not included in the present integrative cross-dataset analysis. The complete list of ChIP-Atlas enrichment results is provided in Supplementary Material S3.

Together, these findings demonstrate that partial reprogramming with Yamanaka factors, regardless of factor combination, induces reproducible yet limited methylation changes across independent datasets and array platforms, suggesting that targeted epigenetic remodeling may contribute to the reported anti-aging and regenerative effects.

## 3. Discussion

To investigate conserved methylation changes, we performed a cross-species integrative secondary analysis of five independent DNA methylation datasets from partial reprogramming experiments with pluripotency-associated transcription factors (OSK, OSKM, and OSKMLN) [10,16,19,33,35,37]. The datasets included samples (*n* = 189 samples, control = 91 samples, and treated = 98 samples) from three mammalian species, including humans, mice, and rats, covering multiple tissues and cell types and integrating diverse partial reprogramming protocols. The analytical workflow integrated rigorous filtering, harmonization, and differential methylation analysis, allowing us to identify CpG sites that are consistently affected by partial reprogramming despite differences in hybridization platform, tissue, species, and treatment conditions. Of the 929,009 CpGs analyzed, only two CpG sites were consistently differentially methylated across all datasets. Two additional CpG sites were present across all genomes and treatment conditions but were not detected in the Olova et al. dataset [16], while the remaining ten CpGs were restricted to rat and mouse genomes (Supplementary Material S1, Tables S1–S4). This finding emphasizes that partial reprogramming induces targeted epigenetic changes within a shared subset of CpGs across mammals and cell types. These loci may likely represent a core epigenetic signature of the reprogramming process, while additional methylation changes may occur in a cell-type-, species-, and treatment-specific manner [40,41], reflecting the biological heterogeneity widely observed in partial and full reprogramming experiments [32,33,34,42].

Functional enrichment analysis of genes associated with the shared differentially methylated CpG loci highlighted biological processes related to cell-cycle regulation, telomere maintenance, developmental patterning and lipid metabolism. Although these findings were based on a limited number of loci, they suggest that the recurrent methylation changes identified across partial reprogramming datasets may affect pathways relevant to the aging process.

Annotation of the individual CpG-associated genes further supported these observations. Several loci mapped onto genes involved in development, transcriptional regulation, metabolism, DNA repair and cellular signaling, supporting prior observations that methylation changes at regulatory CpG sites may contribute to modulation of cellular function and plasticity [6,43,44]. Among these, *HOXA10*, a homeobox transcription factor crucial in embryonic development [45], displayed hypomethylation at its promoter, specifically the TSS200 region. This finding may suggest enhancement of transcriptional plasticity and partial reactivation of developmental programs in a gene that is normally downregulated with age [46]. Similarly, *Irx3*, another homeobox gene [47], and *Ptch1*, a Hedgehog signaling regulator [48], may reflect modulation of regenerative pathways that contribute to the restoration of age-related phenotypes towards a more youthful state [49], in line with proposed mechanisms of rejuvenation [50].

Other genes associated with the location of these CpGs suggest links to metabolic regulation and stress response, both key processes in aging biology. *ACSL6*, involved in long-chain fatty acid metabolism [51], and *Glcci1*, implicated in the glucocorticoid response [52], may participate in metabolic and hormonal pathways affected during aging [53]. *Tmem26*, which plays a role in beige adipocyte differentiation [54], further highlights the potential implication of these epigenetic changes, given that this type of cell population declines with age [55]. CpGs mapping onto *FBXL7* [56], *Rnf19b* [57], and *NABP2* [58] may be involved in protein homeostasis and DNA maintenance, potentially contributing to enhanced cellular resistance against age-related diseases [59,60]. Additional loci, including those mapped onto *Ptprm* [61] or onto intergenic regions near uncharacterized transcripts, may reflect regulatory elements activated by partial reprogramming [62,63]. One CpG site was mapped within AABR07057586.1, an uncharacterized region from the rat genome [64]. Notably, many of these genes have not been highlighted in previous reprogramming studies, which have largely concentrated on transcriptional changes involving classical pluripotency regulators (OSKMLN) or on epigenetic age metrics [16], rather than on consistent epigenetic markers. This suggests that these CpG sites identified here may provide novel insights into the mechanistic basis of partial reprogramming.

The distribution of hyper- versus hypomethylation across promoter, gene body, exon, and intronic regions suggests precise gene regulation rather than broad transcriptional activation or silencing. Hypermethylated sites in gene bodies and exons may influence transcription or alternative splicing [65,66,67,68], whereas hypomethylation at promoters may facilitate increased transcriptional activity [69]. Altogether, these patterns indicate that partial reprogramming selectively targets key regulatory sites that may contribute to regenerative effects. However, this interpretation should be considered in the context of this study’s limitations. In particular, the relatively small number of significant CpG sites identified may reflect constraints related to effect-size heterogeneity, cross-dataset variability, and incomplete CpG overlap across hybridization platforms.

To further characterize the identified loci, we assessed their evolutionary conservation using vertebrate PhyloP scores. The conservation analysis revealed that seven annotated CpGs were located within genomic regions exhibiting moderate to high evolutionary conservation scores. While evolutionary conservation alone does not imply functional significance, the presence of multiple CpGs within conserved genomic regions supports their potential biological relevance. Nevertheless, the variability in conservation scores indicates that recurrent methylation changes are not restricted to highly conserved genomic positions.

To investigate the regulatory context of the identified CpGs, ChIP-Atlas transcription factor enrichment analysis was performed. Although several transcription datasets overlapped with the queried regions, none reached statistical significance. Isolated overlaps with the KLF4, MYC, and NANOG ChIP-seq datasets were identified; these observations were limited to a single location and originated from cellular contexts not represented in the analyzed datasets. Therefore, the present analysis does not provide strong evidence for the preferential localization of the identified CpGs within known binding regions of reprogramming-associated transcription factors.

The question we sought to answer through this study was what impact OSK-mediated partial reprogramming has on the methylome. We identified a restricted set of CpG sites that were consistently and significantly modified following Yamanaka factor expression across species, tissues, and experimental conditions. We tried to go further and look for clues to possible mechanisms but, based on the currently available public data, drawing definitive conclusions would be largely speculative. Nevertheless, these findings provide a focused set of candidate regulatory loci that may guide future experimental validation, including ongoing in vivo studies of OSK-mediated epigenetic reprogramming in aging models.

Our findings may inform future partial reprogramming experiments and support the potential translation of this approach into systemic anti-aging therapy. Notably, epigenetic reprogramming has already reached Phase I clinical trials, but only as a targeted treatment for glaucoma [20]. One of the main limitations regarding a systemic anti-aging treatment remains the complex and heterogeneous nature of aging, including variability in aging rates even in cells within the same tissue [70]. Given that only a small subset of CpGs consistently respond to partial reprogramming, future interventions could be optimized to target these key regulatory loci, potentially improving efficacy and reducing risks associated with overexpression of pluripotency factors, particularly in OSKM or OSKMLN combinations [13,71,72]. The genes identified could serve as biomarkers to optimize treatment duration, dosing, or factor combinations. Moreover, the presence of these conserved CpGs across tissues and cell types suggests that systemic rejuvenation effects may be achievable by targeting only a small number of conserved methylation markers, potentially without the need for additional epigenetic changes across the methylome induced by Yamanaka factors. However, this remains a hypothesis that requires further experimental validation.

Finally, although the overall number of differentially methylated CpGs is small compared with the entire methylome, their functional relevance and evolutionary conservation highlight that targeted epigenetic modulation can have a substantial biological impact. Partial reprogramming appears to refine specific regulatory circuits involved in developmental plasticity, metabolic homeostasis, DNA repair, and proteostasis, providing a mechanistic framework for how limited methylation changes may contribute to cellular rejuvenation and regenerative potential.

## 4. Materials and Methods

### 4.1. Selection of GEO Datasets

All analyses were conducted in R (v. 4.5.2). Data manipulation was performed using tidyverse-based workflows. DNA methylation datasets were retrieved from the Gene Expression Omnibus (GEO) data repository. Datasets were included if they met the following criteria: (*i*) DNA methylation quantification using Illumina’s methylation hybridization arrays, such as Infinium HumanMethylation450K BeadChip (450K) (GPL13534), Infinium HumanMethylationEPIC (EPIC) (GPL21145), or Horvath’s Mammalian Methylation array (GPL28271); (*ii*) availability of old control and partial reprogramming treatment samples (OSK, OSKM, and OSKMLN); and (*iii*) availability of normalized β-values and corresponding sample-level metadata. Five datasets meeting these criteria were included: GSE252401 [73], GSE142439 [74], GSE147436 [75], GSE190665 [76], and GSE54848 [77].

The GSE252401 dataset [10] examined the regenerative and anti-aging effects of AAV-OSKM-induced partial reprogramming in aged rat hippocampi. The GSE142439 dataset [35] investigated partial reprogramming in human dermal fibroblasts and endothelial cells following treatment with mRNA-OSKMLN. The GSE147436 dataset [19] applied an AAV-OSK partial reprogramming protocol to differentiated human SH-SY5Y neurons. GSE190665 [33] involved AAV-OSKM treatment in premature aging mouse models. Finally, the GSE54848 dataset [16,37] consisted of human dermal fibroblasts virally transduced to express OSKM until induced pluripotent stem cell (iPSC) transformation. From the GSE54848 dataset, only samples within the partial reprogramming “safety window”, as defined by Olova et al. [16], were retained. Across all datasets, only untreated control samples and partial-reprogramming-treated samples were included. Samples not relevant to the study objective (young controls or unrelated treatments) were excluded from both β-matrices and associated metadata.

### 4.2. Data Retrieval and Preprocessing

Datasets were downloaded using the GEOquery R package (v.2.78.0). Series matrix files containing normalized β-values were retrieved, along with supplementary processed data files where available. For each dataset, β-values matrices were extracted with Illumina CpG IDs, also known as probe IDs, preserved as row names through systematic curation. Treatment labels (OSK, OSKM, and OSKMLN) were standardized as “treated”, and corresponding untreated samples were labeled “ctrl”. Sample identifiers were aligned between β-values matrices and phenotype tables using exact string matching. Dataset origin was recorded as a covariate to account for study-specific effects in subsequent statistical modeling.

### 4.3. DNA Methylation Data Processing and Quality Control

For statistical analysis, β-values were converted to M-values using the logit transformation:

M = log_2_(β/(1 − β))

To avoid infinite values while converting to M-values, β-values equal to 0 or 1 in the Olova dataset were adjusted by applying an ε factor of 10^−3^. M-values were used for differential methylation analysis due to improved statistical properties and variance stabilization [78].

To enable cross-platform comparability while preserving maximum CpG coverage, we implemented two complementary approaches: (*i*) a union-based approach retaining all CpG ID sites present in any dataset and (*ii*) an intersection-based approach restricted to CpG ID sites common across all five datasets. For the union approach, CpGs with more than 20% missing values (*NAs*) across samples were excluded to ensure robustness. The intersection approach, based on shared Illumina CpG probe identifiers (IllumIDs) retained across successive array generations, was more conservative and yielded a complete matrix without missing values after removing nine samples containing *NAs*. Both strategies were used to assess the consistency of findings under different analytical assumptions. The combined dataset generated using the union approach consisted of 189 samples and 929,009 CpG sites and was used for downstream differential methylation analysis.

### 4.4. Differential Methylation Analysis

Differential methylation analysis was performed using linear modeling implemented in the limma package (v3.66.0). The design matrix included treatment status (treated vs. control) as the primary variable of interest and dataset origin as a covariate to account for batch and platform differences. The linear model was fitted to M-values, followed by empirical Bayes moderation to stabilize variance estimation across CpGs.

For the union-based analysis, we first performed differential methylation on the combined matrix (929,009 CpGs) and then applied quality control by retaining only CpGs with <20% missing data, resulting in 37,554 CpGs for the final analysis. Differentially methylated CpGs were identified using two absolute thresholds: statistical significance (*adj.p.Val* < 0.05) and biological relevance (|log_2_FC| > 0.5). The intersection-based analysis followed the same statistical framework and was applied to the 5306 CpGs shared across all datasets.

### 4.5. Genomic Annotation, CpG Distribution and Functional Enrichment

Significant CpGs from the union analysis (*n* = 14) were annotated using Bioconductor platform-specific annotation packages. For Illumina 450 samples, annotation was performed using the IlluminaHumanMethylation450kanno.ilmn12.hg19 package (v0.6.1), and for EPIC samples, the IlluminaHumanMethylationEPICanno.ilm10b4.hg19 package (v0.6.0) was used. For Horvath Mammalian Methylation samples, annotation for *Rattus norvegicus* probe IDs was obtained from the Mammalian Methylation Consortium repository (Rattus_norvegicus.rnor_6.0.101.HorvathMammalMethylChip40.v1.csv) and was downloaded from the GitHub repository [79]. Given the conserved design of the platform, separate annotation for *Mus musculus* was not required, as the CpG sites were mapped onto the *Rattus norvegicus* reference annotation.

CpGs were classified according to CpG island contexts (island, shore, shelf, or open sea) and gene-related features (promoter regions, exons, introns, or intergenic regions). Based on the direction of effect, CpGs were categorized as hypermethylated (log_2_FC > 0.5; *adj.p.Val* < 0.05) or hypomethylated (log_2_FC < −0.5; *adj.p*.Val < 0.05). Genomic annotation was performed using the reference genome builds corresponding to the Illumina and Horvath Mammalian Methylation Consortium platform annotation files (rn6). Reproducibility was ensured through scripted workflows and consistent pre-processing across datasets.

Functional enrichment analysis of the genes associated with the four CpG loci consistently identified across all analyzed species and cell types was performed using Gene Ontology (GO) Biological Process enrichment analysis.

### 4.6. Evolutionary Conservation and Transcription Factor Enrichment Analysis

Evolutionary conservation was assessed using PhyloP conservation scores obtained from UCSC Genome Browser. Human CpG sites (hg19 assembly) were compared with the “100 Vertebrates Basewise Conservation by PhyloP” track, whereas rodent loci (rn6 assembly) were evaluated using the corresponding “20 Vertebrates Basewise Conservation by PhyloP” track, according to the standard conservation tracks available for each reference genome.

Transcription factor enrichment analysis was performed on the four CpG sites consistently identified across all species and cell types included in the analysis. Genomic regions extending ± 1 kb around each CpG site were analyzed using the Enrichment Analysis tool against the public ChIP-seq dataset collection.

### 4.7. Visualization

For visualization and biological interpretation, statistically significant CpGs (*n* = 14) were merged with their corresponding β-values across all samples. This allowed representation of both effect sizes and methylation level. All visualizations were generated using ggplot2 (v4.0.2). Differential methylation results for all 37,554 CpGs were visualized in a volcano plot displaying log_2_ fold change (M-values) against statistical significance (−log_10_ adjusted *p*-value). A heatmap was generated for hypermethylated (*n* = 8) and hypomethylated (*n* = 6) CpGs using β-values from all 189 samples. Samples were ordered by treatment status (control first, treated second) to highlight group differences. Moreover, for each of the 14 significant CpGs, boxplots were generated to visualize β-value distributions between control and treated groups.

### 4.8. Code Availability and Reproducibility

The complete R script, including step-by-step instructions, is available online in the GitHub repository: https://github.com/Independent-Research-Lab/PartialReprogramming-DNAm-MetaAnalysis/.

## Figures and Tables

**Table 1:** Overview of the GEO DNA methylation dataset used in this analysis, including platform, tissue or cell type, species, factors used for the induction of partial reprogramming, and sample distribution.

Dataset	GEO Accession	Platform	Tissue or Cell Type	Treatment	Samples		Reference
					Control	Treatment	
1	GSE252401	Mammal40k	Hippocampus (*Rattus norvegicus*)	OSKM	6	8	[10]
2	GSE142439	EPIC	Fibroblasts and endothelial (*Homo sapiens*)	OSKMLN	8	8	[35]
3	GSE147436	EPIC	SH-SY5Y (*Homo sapiens*)	OSK	3	3	[19]
4	GSE190665	Mammal40k	Liver, spleen, skeletal muscle, lung, kidney, and skin (*Mus musculus*)	OSKM	71	73	[33]
5	GSE54848	450K	Fibroblasts (*Homo sapiens*)	OSKM	3	6	[16,37]

**Table 2:** Genomic annotations of the 14 significantly differentially methylated CpG sites following partial reprogramming.

CpG	Chr	Position	Gene	Gene_Region	Platform	Methylation Status
cg20011459	chr5	15936642	*FBXL7*	Body	450K	Hypomethylated
cg01397139	chr7	27213966	*HOXA10*	TSS200; Body	450K	Hypomethylated
cg20072785	chr5	131312398	*ACSL6*	ExonBnd	EPIC	Hypermethylated
cg03201458	chr12	56618645	*NABP2*	Body	EPIC	Hypermethylated
cg00992842	chr4	34610198	*Glcci1*	Exon	Horvath	Hypermethylated
cg02586754	*NA*	*NA*	*NA*	*NA*	Horvath	Hypermethylated
cg04834810	chr5	147267762	*Rnf19b*	Exon	Horvath	Hypermethylated
cg06347432	chr9	16744865	*Srf*	Exon	Horvath	Hypomethylated
cg12379857	chr19	15909024	*Irx3*	Intergenic_upstream	Horvath	Hypomethylated
cg17604804	chr17	1045326	*Ptch1*	Intron	Horvath	Hypomethylated
cg18665094	chr9	115024612	*Ptprm*	Intron	Horvath	Hypomethylated
cg21884043	*NA*	*NA*	*NA*	*NA*	Horvath	Hypermethylated
cg23426225	chr20	21563729	*Tmem26*	Intergenic_downstream	Horvath	Hypermethylated
cg23704142	chr7	79334278	AABR07057586.1	Intergenic_downstream	Horvath	Hypermethylated

Chr = chromosome, Position = genomic coordinate of the CpG, *FBXL7* = F-box and leucine-rich repeat protein 7, *HOXA10* = homeobox A10, *ACSL6* = acyl-CoA synthetase long-chain family member 6, *NABP2* = nucleic-acid-binding protein 2, *Glcci1* = glucocorticoid-induced 1, *Rnf19b* = ring-finger protein 19B, *Srf* = serum response factor, *Irx3* = iroquois homeobox 3, *Ptch1* = patched 1, *Ptprm* = protein tyrosine phosphatase receptor type M, *Tmem26* = transmembrane protein 26, AABR07057586.1 = gene identifier from rat genome assemblies, Gene_region = CpG location relative to gene, and *NA* = not available.

## Data Availability

The necessary instructions, R functions, and complete code to reproduce our analysis are available in our public GitHub repository: https://github.com/Independent-Research-Lab/PartialReprogramming-DNAm-MetaAnalysis/.

## Supplementary Materials

The following supporting information can be downloaded at https://www.mdpi.com/article/10.3390/epigenomes10030055/s1. Supplementary Materials S1: Tables S1–S5. Table S1: Significant CpG sites in treated vs. control samples after union analysis; Table S2: Significant CpG sites in treated vs. control samples after union analysis (with β-values); Table S3: Annotated significant CpGs following union analysis; Table S4: Significant CpG sites in treated vs. control samples after intersection analysis; Table S5: Gene Ontology (GO) Biological Process enrichment analysis of genes associated with shared differentially methylated CpG sites following partial reprogramming; Supplementary Material S2: PhyloP conservation scores for conserved differentially methylated CpG sites; Supplementary Material S3: Transcription factor enrichment analysis of shared CpG loci.
